# Percutaneous Endoscopic Gastrostomy: Mortality and Risk Factors for Survival

**DOI:** 10.4021/gr402w

**Published:** 2012-01-20

**Authors:** Akin Onder, Murat Kapan, Zulfu Arikanoglu, Mesut Gul, Remzi Bestas, Yilmaz Palanci, Haktan Karaman, Bilsel Bac

**Affiliations:** a Department of Surgery, Dicle University Faculty of Medicine, 21280, Diyarbakir, Turkey; b Department of Gastroenterology, Dicle University Faculty of Medicine, 21280, Diyarbakir, Turkey; c Department of Public Health, Dicle University Faculty of Medicine, 21280, Diyarbakir, Turkey; d Departmant of Anesthesia and Reanimation, Dicle University Faculty of Medicine, 21280, Diyarbakir, Turkey

**Keywords:** Percutaneous endoscopic gastrostomy, Morbidity, Mortality, Albumin level

## Abstract

**Background:**

The present study evaluated long-term risk factors for survival in patients who have undergone Percutaneous endoscopic Gastrostomy, as well as morbidity and mortality rates.

**Methods:**

The retrospective study included 44 patients who underwent placement of a percutaneous endoscopic gastrostomy tube at various departments at Dicle University Medical Faculty between April 2008-September 2010.

**Results:**

The study evaluated 23 women (52.3%) and 21 men (47.7%), with a median age of 50 ± 20 (17 - 87) years. Median time for Percutaneous endoscopic Gastrostomy placement was 23 ± 8.3 (5 - 45) minutes per patient. Total morbidity was 15.9%, including wound infection (4), tube occlusion (1), peristomal leakage (1), and abdominal wall bleeding (1). Short-term complications were not associated with albumin level (P = 0.312).The median hospital stay was 49.34 ± 60.99 (1 - 314) days. The mean follow-up period was 13.07 ± 13.12 (1 - 41) months. The above-normal level of albumin was found to be effective on survival (P = 0.024). Mortality occurred in 18 (40.9%) patients during the follow-up.

**Conclusions:**

Percutaneous endoscopic Gastrostomy is both safe and effective in that it does not require surgical operation and it can be performed under surface anesthesia. The serum albumin level with patients who have undergone percutaneous endoscopic gastrostomyis an effective factor for survival.

## Introduction

There have been many methods used to provide enteral nutrition for patients who have normal gastrointestinal functions but do not maintain adequate oral nutrition. Among these are percutaneous endoscopic gastrostomy (PEG), nasogastric tube, laparoscopic or surgical gastrostomy, and percutaneous fluoroscopic jejunostomy. PEG was first introduced by Gauderer and Ponsky in 1980 [1]. Once the indications are diagnosed, PEG tube could be inserted through various methods. “Pull” method is the most viable one [2]. Compared to surgical gastrostomy, PEG insertion is a cheaper, more practical and less risky method which could be performed on the patient’s bed–without needing the endoscopy unit–and which only requires intravenous sedation and local anesthesia [3]. In addition to enteral nutrition, PEG insertion also provides gastrointestinal decompression [4]. There have been numerous studies analyzing PEG-related morbidity as well as prognostic factors [5]. The present study evaluated long-term risk factors for survival in patients who have undergone PEG, as well as PEG-related morbidity and mortality rates.

## Materials and Methods

The retrospective study consisted of 44 patients who underwent placement of a PEG tube at various departments at Dicle University Medical Faculty between April 2008-September 2010. PEG tube insertion was undertaken in patients who had no or insufficient oral intake for at least 4 - 6 weeks, who had been fed by nasogastric for more than one month, and who were not in the terminal period [6, 7]. Patients were excluded if they had undergone surgical gastrostomy, or had previously received PEG insertion or tube replacement. Most patients were below 8 in Glascow coma scale and were receiving mechanical ventilation. The other patients who underwent PEG had poor swallowing functions although they remained conscious. Age, gender, primary diseases, associated diseases, laboratory parameters, PEG placement time, hospital stays, morbidity, mortality, and survival periods were analyzed. Normal hemostasis was accepted as the prerequisite. The procedure was performed at the bedsides in all departments. All the processes were accompanied by surface anesthesia (midazolam, ketamine, and diprivan) and monitored. Before insertion, patients were examined via endoscopy to investigate the possibility of obstruction in their upper gastrointestinal channel or lesion on the anterior gastric wall. The process was administered by two doctors (one for endoscopic interference and the other for percutaneous interference). Percutaneous interference was performed through the epigastrium, and sterilization instructions were obeyed while the endoscopic light that is visible through the skin or manual fluctuation was used. Following the procedure, carers were taught cleaning techniques for the tube and the surrounding region. No prophylactic antibiotics were given except for patients with primary diseases. Patients were fasted for 12 hours before PEG insertion, and following the fluid support, the procedure was performed with the aid of Olympus GIF XQ-240 video endoscope by pulling the guide wire (the pull method).The antrum-fundus point was chosen as the ideal region. The procedure was undertaken with PEG kit (Boston Scientific, France). After PEG insertion, once no complications like abdominal pain or peristomal leakage were detectedby means of the 20 ml of water given 24 hours after insertion, patients were routinely fed with 10 cc, which attained desirable level in 7 - 10 days as a result of gradual increase. In addition to oral solutions available in PEG kits, patients were also given more economical gavage-type formulas prepared by families. Also, the medication that should be taken orally can be applied uneventfully by PEG. Patients were followed up in the first week and in the first month after insertion. The patients discharged were checked on the phone. Following the first thirty days, they were visited when they had any problem or routinely in every three months. Laboratory parameters were analyzed at each visit. For serum albumin level < 3 gr/dl was regarded as hypoalbuminemia, while ≥ 3 gr/dl was considered normal. Serum albumin levels became normal in the 6th month in the follow-up period. Mortalities occurred both in short-term (within the first month) and long-term (after the first month). Families were provided with phone numbers which they could reach 24 hours a day, along with the names of doctors in charge, so that they could make contact for any problem or complication.

### Statistical analysis

Data analysis was performed with SPSS 13.0 (SPSS Inc., Chicago, IL, USA). Quantitative values were presented as mean ± Standard deviation. Student-t test was used both for group comparisons and parametric data. For different categories, chi-square test was used. Risk factors for survival were evaluated by using logistic regression test. Survival rates were analyzed by Kaplan-Meier survival curve, and differences between the groups were assessed with the log-rank test. P < 0.05 was regarded significant, and odds ratio (OR) was calculated for each variant.

## Results

**Figure 1:**
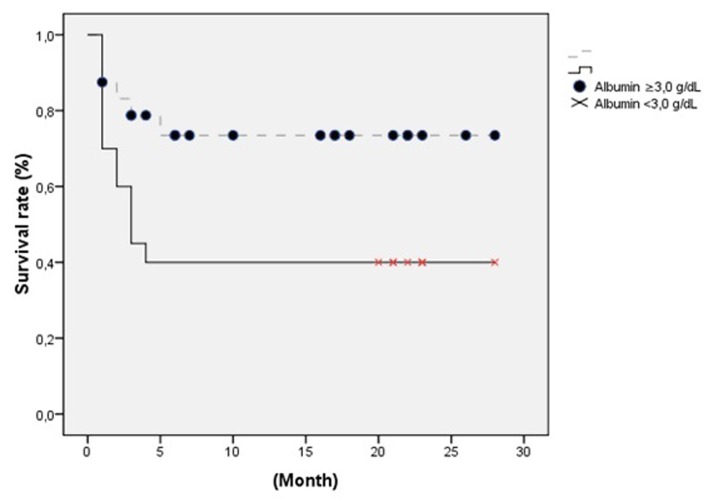
Albumin as a risk factor for survival.

The 44 patients comprised 23 women (52.3%) and 21 (47.7%) men, with a median age of 50 ± 20 (17 - 87) years. The Anesthesiology and Reanimation Department had the highest number of placement with 31 (70.5%) tubes. Common etiologies included cerebrovascular diseases (stroke (17), cerebral hemorrhage (10), intracerebral hematoma (4), and subarachnoid hemorrhage (3)) and neurodegenerative diseases (amyotrophic lateral sclerosis (3), and myotonic dystrophy (1)). Patients categorized by departments and diagnoses are shown in Table 1. Coronary artery disease (CAD) was evident in 5, diabetes melittus (DM) in 3, and chronic obstructive pulmonary disease (COPD) in 2 patients. Pre-PEG laboratory studies revealed hemoglobin and albumin levels as 13.4 ± 2.4 (10 - 16) and 2.85 ± 65.5 (1.50 - 4.43) gr/dl, respectively. Demographic and laboratory data are shown in Table 2. All the patients were using nasogastric catheter and feeding through enteral access. Prior to PEG insertion, 28 patients underwent tracheostomy. The average length of time per procedure was 23 ± 8.3 (5 - 45) minutes. Total morbidity occurred in 15.9% after PEG insertion. Wound infection was the most common complication, as shown in Table 3. Patients with low albumin level had a complication rate of 23.1%, while the ones with normal level had a rate of 11.1%. Short-term complications were not associated with albumin level (P = 0.312). PEG tube was removed in one patient who restarted oral intake. The patient had a gastrocutaneous fistula which healed uneventfully in 3 - 4 days. No mortality occurred regarding postoperative procedures. Total mortality occurred in 18 (40.9%) patients because of non-procedural causes.Inthe short-term follow-up, mortality developed in 8 (18.2%) patients.Of these, 3 died from cardiac arrest, 2 from pneumonia, and 3 from primary diseases. During the long-term, 10 patients developed mortality, of which 2 died from cardiac arrest, 2 from pneumonia, and 6 from primary diseases. The median hospital stay was 49.34 ± 60.99 (1 - 314) days. The mean follow-up period was 13.07 ± 13.12 (1 - 41) months. In univariate logistic regression analysis, the serum albumin level during follow-up was associated with survival (OR: 4.5, Cl = 1.24 - 16.28, P = 0.022), as shown with other variants in Table 4. This association was also approved by multivariate logistic regression analysis (OR: 4.09, Cl = 1.08 - 15.5, P = 0.038), as shown with other variants in Table 5. Risk factors for surviving patients were analyzed by Kaplan-Meier curve. Albumin as a risk factor for survival is shown in Figure 1.

**Table 1:** Distribution of Patients as Regards Their Departments and Diagnoses

	N	%
Department		
anesthesia	31	70.5
neurology	5	11.4
Gastroenterology	3	6.8
Neurosurgery	2	4.5
General surgery	2	4.5
Infectious Diseases and Clinical Microbiology	1	2.3
Diagnosis		
Cerebral vascular diseases	34	77.3
Stroke	17	
Cerebral hemorrhage	10	
Intracerebral hemorrhage	4	
Subaracnoid bleeding	3	
neurodegenerative Diseases	4	9
Amiyotropik lateral sclerosis	3	
Myotonic dystrophy	1	
malignancy	4	9
dementia	1	2.3
after surgery	1	2.3

**Table 2:** Patients’ Demographic and Laboratory Data

Age (years)	50 ± 20 (17 - 87)
Female/male	23/21
WBC (/mm^3^)	10900.1 ± 4000.7(4.300 - 17.000)
Lymphocytes(/µL )	1587 ± 916 (575 - 5580)
Hb (g/dL)	13.4 ± 2.4 (10 - 16),
Alb (g/dL)	2.85 ± 65.5 (1.50 - 4.43)

**Table 3:** Postoperative Complications

	N	%
Wound enfection	4	9
Periostomal leak	1	2.3
Tube occlusion	1	2.3
Hemorrhage	1	2.3

**Table 4:** Univariate Analysis in Patients Without Malignant Diseases of Risk Factors for Survival After PEG Placemen

	Odds ratio	CI 95%	P
Serum albumin level g/dL	4.5	1.24 - 16.28	0.022
Serum lymphocytes count/µL	0.89	0.25 - 3.22	0.860
Age (years)	1.2	0.27 - 5.26	0.810
Gender	0.37	0.11 - 1.28	0.116
Complication with PEG placement	0.84	0.17 - 4.07	0.828
Cerebrovascular diseases	0.52	0.14 - 1.91	0.328

**Table 5:** Multivariate Analysis in Patients Without Malignant Diseases of Risk Factors for Survival After PEG Placement

	Odds ratio	CI 95%	P
Serum albumin level g/dL	4.09	1.08 - 15.5	0.038
Serum lymphocytes count /µL	1.70	0.41 - 7.13	0.462
Serum sodium level meq/L	0.32	0.73 - 1.41	0.320

## Discussion

Maintaining adequate nutrition is a key issue for treatment protocols in intensive care, particularly in critical cases [8, 9]. Enteral nutrition is aimed to sustain mucosal barrier function along with intestinal immune response and normal flora by preserving gastrointestinal mucosal integrity [10]. As its success relies on the health personnel and it is liable to result in metabolic disorders, long-term usage of parenteral nutrition is not recommended for patients with chronic neurological disorders; for those with serious head and neck traumas; and for those who underwent upper respiratory surgery; as well as for cases with no oral intake. For these patient groups, PEG is recommended as a means of enteral nutrition. Cerebrovascular diseases are reported as the most common diseases, as evident in 65.9 - 75.3% of patients who undertake PEG insertion [5, 11, 12]. We found them in 77.3% of our patients. The length of time for PEG placement procedure is reported as 7.5 - 34 minutes [5, 13]. We had an average of 23 minutes.

Procedural complications are likely to occur both during and after PEG insertion. Common complications during the insertion are pneumoperitoneum, abdominal wall bleeding, colon trauma, liver or splenic laceration, and intra-retroperitoneal bleeding. Unless there is additional peritonitis, pneumoperitoneum goes unnoticed by itself as it retreats in 72 hours although it is evident at a rate of 50 - 85% [14, 15]. Abdominal wall bleeding is generally caused by vein injuries during PEG tube insertion, but it can be taken under control via tight compression [16]. Liver and splenic injuries, which are caused by careless penetration of the syringe into locations outside the stomach, are relatively rare. Intraabdominal and retroperitoneal bleedings are possible cases secondary to liver injuries [17, 18]. We found no peritonitis secondary to pneumoperitoneum in our patients. However, it is possible that some moderate cases of pneumoperitoneummay have been overlooked in our patients since the procedures took place in intensive care units and they were undertaken in indigent patients. Abdominal wall bleeding occurred in one patient. The patient was receiving low molecular weight heparin because of cerebrovascular disease. Bleeding was stopped by compression in the 48th hour. Wound infection is the most common postoperative complication. Development rate for patients with no prophylaxis application and for those with prophylactic antibiotics are 18% and 3%, respectively [19]. Othercommon complications include peristomal pain, tube occlusion, peristomal leakage, and aspiration. Tube occlusion is a possible result of drugs which are applied to large volume enteral nutritional products without decomposition or melting in water. In order to prevent occlusion, nutritional tubes should be cleaned with 30 - 60 ml of water in every 4 hours [20]. Aspiration is more common in patient groups of sedation, old age, and neurological diseases [21]. Peristomal leakage generally occurs in the first several days after procedure. It is more common in patients with poor wound healing and poor tissue nutrition, and/or in DM patients [22]. Reportshold it that wound healing problems are likely to occur in DM patients as a result of decline in adherence, phagocytosis and chemotaxis of leukocytes, and that these functions are corrected via hyperglycemia regulation [23, 24].

Wound infection was confirmed as the most common complication in our patients. No prophylactic antibiotic was given to our patients, except for the ones who received antibiotics because of primary diseases. DM was present in 2 of the patients who developed wound infection. Hyperglycemia-associated infection was treated by daily dressing and antibiotics. Occlusion in the tube was removed by intermittent washing. The patient in our study, who developed peristomal leaking, had severe malnutrition. For the patient, total parenteral nutrition was added to enteral nutrition.

There have been many prognostic factors reported for patients with PEG insertion. Among them are ageing, gender, dementia, low serum albumin level, low cholesterol level, hyponatremy, complicated pneumonia, cardiovascular diseases, and malignant diseases [5, 25-27].

Despite its sensitivity, specificity, lowness, and its fast turnover, albumin is a risk factor for survival in patients with PEG insertion [25, 28]. Though not effective on postoperative morbidity, albumin level was confirmed as a risk factor for survival in our study, where patients with malignant diseases were not included.

Lymphocyte count was another parameter which was studied as a risk factor for survival in our study. However, it was reported as a risk factor for long-term survival as the decreasing number of lymphocyte is resulted from the increasing number of T-suppressor cells in patients with malignant diseases [29]. Nonetheless, we did not find lymphocyte count as significant in our study, likely because of low malignity rate and longer survival periods. It was not significant for survival in benign diseases either.

Mortality is reported as 1 - 3% in PEG procedures, while it could be as high as 15% in open gastrostomies [30, 31]. No mortality as relating PEG procedures was reported in our study. The mortalities that were caused by non-procedural reasons were reported as 8.2 - 32.8% for the short-tem (the first 30 days), and 38 - 90% for the long-term (one year) [11, 32-34]. In the short-term, 8 mortalities occurred. Of these, 37.5% died from primary diseases (cerebrovascular), 37.5% from associated diseases (CAD and COPD), and 25% from pneumonia. As for the long-term, 10 (22.7%) mortalities occurred. Of these, 60% had primary diseases (3 with malignant diseases and 3 with cerebrovascular diseases), 20% had associated diseases (CAD and COPD), while 20% had pneumonia.

Complicated pneumonia was previously known as a risk factor for survival, whereas it has been outlawed as a risk factor due to latest developments and new precautions taken against hospital-bound infections.

The limitation of this study adheres to the failure of analyzing other parameters that confirm malnutrition, largely because of the low number of patients and the retrospective nature of the study.

In conclusion, PEG is both safe and effective for patients who have not maintained adequate oral intake for a long time as it does not require surgical operation and it can be performed under surface anesthesia. It leads to low morbidity rate which could be easily treated. Albumin at above-normal level was found to be effective on survival during long-term follow-up, although it is not effective on morbidity. To avoid malnutrition, PEG should be promptly and effectively inserted in patients with inadequate oral intake.

## References

[1] Gauderer MW, Ponsky JL, Izant RJ (1980). Gastrostomy without laparotomy: a percutaneous endoscopic technique. J Pediatr Surg.

[2] Hiki N, Maetani I, Suzuki Y, Washizawa N, Fukuda T, Yamaguchi T (2008). Reduced risk of peristomal infection of direct percutaneous endoscopic gastrostomy in cancer patients: comparison with the pull percutaneous endoscopic gastrostomy procedure. J Am Coll Surg.

[3] Dwyer KM, Watts DD, Thurber JS, Benoit RS, Fakhry SM (2002). Percutaneous endoscopic gastrostomy: the preferred method of elective feeding tube placement in trauma patients. J Trauma.

[4] Schrag SP, Sharma R, Jaik NP, Seamon MJ, Lukaszczyk JJ, Martin ND, Hoey BA (2007). Complications related to percutaneous endoscopic gastrostomy (PEG) tubes. A comprehensive clinical review. J Gastrointestin Liver Dis.

[5] Tokunaga T, Kubo T, Ryan S, Tomizawa M, Yoshida S, Takagi K, Furui K (2008). Long-term outcome after placement of a percutaneous endoscopic gastrostomy tube. Geriatr Gerontol Int.

[6] Chong VH, Vu C (2006). Percutaneous endoscopic gastrostomy outcomes: can patient profiles predict mortality and weaning?. Singapore Med J.

[7] Stroud M, Duncan H, Nightingale J (2003). Guidelines for enteral feeding in adult hospital patients. Gut.

[8] Kudsk KA, Croce MA, Fabian TC, Minard G, Tolley EA, Poret HA, Kuhl MR (1992). Enteral versus parenteral feeding. Effects on septic morbidity after blunt and penetrating abdominal trauma. Ann Surg.

[9] Moore FA, Feliciano DV, Andrassy RJ, McArdle AH, Booth FV, Morgenstein-Wagner TB, Kellum JM (1992). Early enteral feeding, compared with parenteral, reduces postoperative septic complications. The results of a meta-analysis. Ann Surg.

[10] Marik PE, Zaloga GP (2001). Early enteral nutrition in acutely ill patients: a systematic review. Crit Care Med.

[11] Erdil A, Saka M, Ates Y, Tuzun A, Bagci S, Uygun A, Yesilova Z (2005). Enteral nutrition via percutaneous endoscopic gastrostomy and nutritional status of patients: five-year prospective study. J Gastroenterol Hepatol.

[12] Verhoef MJ, Van Rosendaal GM (2001). Patient outcomes related to percutaneous endoscopic gastrostomy placement. J Clin Gastroenterol.

[13] Kang WM, Yu JC, Ma ZQ, Liu XH (2008). [Clinical application of percutaneous endoscopic gastrostomy/jejunostomy in critically ill patients]. Zhongguo Yi Xue Ke Xue Yuan Xue Bao.

[14] Wiesen AJ, Sideridis K, Fernandes A, Hines J, Indaram A, Weinstein L, Davidoff S (2006). True incidence and clinical significance of pneumoperitoneum after PEG placement: a prospective study. Gastrointest Endosc.

[15] Blum CA, Selander C, Ruddy JM, Leon S (2009). The incidence and clinical significance of pneumoperitoneum after percutaneous endoscopic gastrostomy: a review of 722 cases. Am Surg.

[16] Seidner DL, Ghanta RK (2005). Management of a traumatic gastric ulcer with a low-profile gastrostomy tube. Nutr Clin Pract.

[17] Wiggins TF, Kaplan R, DeLegge MH (2007). Acute hemorrhage following transhepatic PEG tube placement. Dig Dis Sci.

[18] Lau G, Lai SH (2001). Fatal retroperitoneal haemorrhage: an unusual complication of percutaneous endoscopic gastrostomy. Forensic Sci Int.

[19] Ahmad I, Mouncher A, Abdoolah A, Stenson R, Wright J, Daniels A, Tillett J (2003). Antibiotic prophylaxis for percutaneous endoscopic gastrostomy—a prospective, randomised, double-blind trial. Aliment Pharmacol Ther.

[20] Mathus-Vliegen LM, Koning H (1999). Percutaneous endoscopic gastrostomy and gastrojejunostomy: a critical reappraisal of patient selection, tube function and the feasibility of nutritional support during extended follow-up. Gastrointest Endosc.

[21] Safadi BY, Marks JM, Ponsky JL (1998). Percutaneous endoscopic gastrostomy. Gastrointest Endosc Clin N Am.

[22] McClave SA, Chang WK (2003). Complications of enteral access. Gastrointest Endosc.

[23] Lee JK, Kim JJ, KİM YH, Jang JK, Son HJ, Peck KR (2002). İncreased risk of periostomal wound infection after percutaneous endoscopic gastrostmy in patients with diabetes mellitus. Digest Liver Dis.

[24] Clement S, Braithwaite SS, Magee MF, Ahmann A, Smith EP, Schafer RG, Hirsch IB (2004). Management of diabetes and hyperglycemia in hospitals. Diabetes Care.

[25] Nair S, Hertan H, Pitchumoni CS (2000). Hypoalbuminemia is a poor predictor of survival after percutaneous endoscopic gastrostomy in elderly patients with dementia. Am J Gastroenterol.

[26] Poulsen M, Trezza M, Atimash GH, Sorensen LT, Kallehave F, Hemmingsen U, Jorgensen LN (2009). Risk factors for morbidity and mortality following gastroenterostomy. J Gastrointest Surg.

[27] Janes SE, Price CS, Khan S (2005). Percutaneous endoscopic gastrostomy: 30-day mortality trends and risk factors. J Postgrad Med.

[28] Tominaga N, Shimoda R, Iwakiri R, Tsuruoka N, Sakata Y, Hara H, Hayashi S (2010). Low serum albumin level is risk factor for patients with percutaneous endoscopic gastrostomy. Intern Med.

[29] Satomi A, Murakami S, Ishida K, Mastuki M, Hashimoto T, Sonoda M (1995). Significance of increased neutrophils in patients with advanced colorectal cancer. Acta Oncol.

[30] Eryilmaz MA, Erden V, Memmi N, Basaranoglu G, Celebi F (2002). [Evaluation of percutaneous endoscopic gastrostomy and results]. Ulus Travma Derg.

[31] Cortez-Pinto H, Correia AP, Camilo ME, Tavares L, De Moura MC (2002). Long-term management of percutaneous endoscopic gastrostomy by a nutritional support team. Clin Nutr.

[32] Sanders DS, Carter MJ, D'Silva J, James G, Bolton RP, Bardhan KD (2000). Survival analysis in percutaneous endoscopic gastrostomy feeding: a worse outcome in patients with dementia. Am J Gastroenterol.

[33] Callahan CM, Haag KM, Weinberger M, Tierney WM, Buchanan NN, Stump TE, Nisi R (2000). Outcomes of percutaneous endoscopic gastrostomy among older adults in a community setting. J Am Geriatr Soc.

[34] Mitchell SL, Tetroe JM (2000). Survival after percutaneous endoscopic gastrostomy placement in older persons. J Gerontol A Biol Sci Med Sci.

